# Evolution patterns of *NBS* genes in the genus *Dendrobium* and *NBS*-*LRR* gene expression in *D*. *officinale* by salicylic acid treatment

**DOI:** 10.1186/s12870-022-03904-2

**Published:** 2022-11-14

**Authors:** Jiapeng Yang, Caijun Xiong, Siyuan Li, Cheng Zhou, Lingli Li, Qingyun Xue, Wei Liu, Zhitao Niu, Xiaoyu Ding

**Affiliations:** 1 College of Life Sciences, Nanjing Normal University, Nanjing, 210023 China; 2 Jiangsu Provincial Engineering Research Center for Technical Industrialization for Dendrobiums, Nanjing, 210023 China

**Keywords:** *NBS* genes, *Dendrobium officinale*, ETI system, Salicylic acid

## Abstract

**Background:**

*Dendrobium officinale* Kimura et Migo, which contains rich polysaccharides, flavonoids and alkaloids, is a Traditional Chinese Medicine (TCM) with important economic benefits, while various pathogens have brought huge losses to its industrialization. *NBS* gene family is the largest class of plant disease resistance (*R*) genes, proteins of which are widely distributed in the upstream and downstream of the plant immune systems and are responsible for receiving infection signals and regulating gene expression respectively. It is of great significance for the subsequent disease resistance breeding of *D*. *officinale* to identify *NBS* genes by using the newly published high-quality chromosome-level *D*. *officinale* genome.

**Results:**

In this study, a total of 655 *NBS* genes were uncovered from the genomes of *D*. *officinale*, *D*. *nobile*, *D*. *chrysotoxum*, *V*. *planifolia*, *A*. *shenzhenica*, *P*. *equestris* and *A*. *thaliana*. The phylogenetic results of CNL-type protein sequences showed that orchid *NBS-LRR* genes have significantly degenerated on branches a and b. The *Dendrobium NBS* gene homology analysis showed that the *Dendrobium NBS* genes have two obvious characteristics: type changing and NB-ARC domain degeneration. Because the *NBS-LRR* genes have both NB-ARC and LRR domains, 22 *D*. *officinale NBS-LRR* genes were used for subsequent analyses, such as gene structures, conserved motifs, *cis*-elements and functional annotation analyses. All these results suggested that *D*. *officinale NBS-LRR* genes take part in the ETI system, plant hormone signal transduction pathway and Ras signaling pathway. Finally, there were 1,677 DEGs identified from the salicylic acid (SA) treatment transcriptome data of *D. officinale*. Among them, six *NBS-LRR* genes (*Dof013264*, *Dof020566*, *Dof019188*, *Dof019191*, *Dof020138* and *Dof020707*) were significantly up-regulated. However, only *Dof020138* was closely related to other pathways from the results of WGCNA, such as pathogen identification pathways, MAPK signaling pathways, plant hormone signal transduction pathways, biosynthetic pathways and energy metabolism pathways.

**Conclusion:**

Our results revealed that the *NBS* gene degenerations are common in the genus *Dendrobium*, which is the main reason for the diversity of *NBS* genes, and the *NBS-LRR* genes generally take part in *D*. *officinale* ETI system and signal transduction pathways. In addition, the *D*. *officinale NBS-LRR* gene *Dof020138*, which may have an important breeding value, is indirectly activated by SA in the ETI system.

**Supplementary Information:**

The online version contains supplementary material available at 10.1186/s12870-022-03904-2.

## Background

*Dendrobium*, one of the largest genera in Orchidaceae, is widely distributed in tropical Asia, Australasia, Australia and New Zealand [1, 2]. There are about 120 *Dendrobium* species in China, which are epiphytic on rocks and tree trunks and distributed at high elevations up to 1,200 m [2]. *Dendrobium* orchids, which have accumulated high content of medicinal ingredients [2], are important commercial crops in China because of their horticultural and medicinal values [3]. For example, *Dendrobium officinale* Kimura et Migo, one of the most valuable Traditional Chinese Medicines (TCMs), is rich in polysaccharides, flavonoids and alkaloids [4, 5]. Because of the great demand and the lack of wild resources, industrial cultivation of *D*. *officinale* has been actively promoted in Anhui, Zhejiang, Jiangsu and Guizhou provinces. However, the invasions of pathogens, such as orchid fleck virus, *Dendrobium vein necrosis closterovirus*, *Fusarium oxysporum* and *Fusarium kyushuense*, have led to production reduction, which resulted in great losses for enterprises [6–9]. Therefore, it is important to identify disease resistance (*R*) genes and explore the metabolic pathways of resistance to biotic stress based on the *D*. *officinale* genome.

The plants have evolved pathogen-associated molecular patterns triggered immunity (PTI) and effector-triggered immunity (ETI) systems to defend against the infections of pathogens [10–12]. Among the two systems, PTI will be triggered when the pathogens break through the plant epidermis [13]. With some pathogens bypassing the PTI system, the plants gradually evolved the ETI system, which can recognize specific pathogen effectors, to counter pathogen infection [13, 14]. Plant *R* genes, of which approximately 80% belong to the *NBS* gene family, are the major component of the ETI system [15–18]. NBS proteins are composed of two main domains: (1) nucleotide binding sites (NB-ARCs) domain, which can bind ATP/GTP molecular; (2) C-terminal leucine-rich repeats (LRRs) domain, which recognizes pathogen effectors [19]. *NBS* genes that retained both the NB-ARC domain and the LRR domain were named *NBS-LRR* genes because part of *NBS* genes lacked the variable LRR domain. Based on the different types of domains in the N terminus, the *NBS-LRR* genes are divided into three subfamilies: TIR-NBS-LRR (TNL), CC-NBS-LRR (CNL) and RPW8-NBS-LRR (RNL) in angiosperm [19]. For example, the rice CNL-type NBS proteins RGA5 and RGA4 can directly bind to *Magnaporthe oryzae* effectors Avr-Pia and Avr1-Co39 [14, 20]. Shao et al. used 22 angiosperm genomes to identify different types of *NBS-LRR* genes, the results of which showed that the number of CNL-type *NBS-LRR* genes (*CNL* genes) was greater than TNL-type and RNL-type, and no TNL-type *NBS-LRR* gene was identified in monocots [18]. In grass species, the distribution of *NBS-LRR* genes in chromosomes shows high aggregation and duplication due to local duplications [17]. In conclusion, *NBS* genes, as important components of the plant immune systems, are abundant and widely distributed on different chromosomes.

Recently, with the rapid development of third-generation sequencing technology (PacBio and Nanopore), three chromosomal-level genomes were published in the genus *Dendrobium*, including *D*. *officinale*, *D*. *chrysotoxum* and *D*. *nobile* [2, 21, 22]. The high-quality *D*. *officinale* genome was 1.23 Gb, with a contig N50 value of 1.44 Mb, and 93.53% of contig sequences were anchored to 19 pseudochromosomes [2]. These high-quality *Dendrobium* genomes make it possible for researchers to explore the evolution of *Dendrobium NBS* genes and uncover molecular pathways of the *D*. *officinale* immune systems.

In this study, the *NBS* genes were identified in six orchids and *Arabidopsis thaliana*, and the homologous genes of all *Dendrobium NBS* genes were speculated based on the chromosomal-level genomes. The structure features and *cis*-elements of the *NBS-LRR* genes in *D*. *officinale* were analyzed. Finally, based on the *D*. *officinale* genome, transcriptome analysis was performed on the coding genes by the salicylic acid (SA) treatment. The aims of this study were: (1) to investigate the evolutionary patterns of *NBS* genes in the genus *Dendrobium*; (2) to explore the molecular pathways involved in *D*. *officinale* immune systems; (3) to reveal the response process of *D*. *officinale* immune systems by the SA treatment. We believed that this study will provide us with a comprehensive understanding of the *NBS* gene evolution in *Dendrobium* and the molecular pathways of *D*. *officinale* immune systems.

## Results

### Classification of *NBS* genes in orchidaceae

From the results of Conserved Domain, Pfam and SMART websites, there were 655 putative *NBS* genes identified in six orchids and *A. thaliana* (74 genes in *D*. *officinale*, 169 genes in *D*. *nobile*, 118 genes in *D*. *chrysotoxum*, 57 genes in *P*. *equestris*, 12 genes in *V*. *planifolia*, 15 genes in *A*. *shenzhenica*, and 210 genes in *A*. *thaliana*) (Table 1, Table S5). The 655 *NBS* genes were classified into two subclasses: the NBS-LRR subclass (*NBS-LRR* genes), the proteins of which contain both NB-ARC and LRR domains, and the non-NBS-LRR subclass, the proteins of which lose the LRR domain. Among the *NBS-LRR* genes, the most abundant genes were the CNL-type (10 genes in *D*. *officinale*, 18 genes in *D*. *nobile*, 14 genes in *D*. *chrysotoxum*, 7 genes in *P*. *equestris*, 2 genes in *V*. *planifolia*, 4 genes in *A*. *shenzhenica*, and 40 genes in *A*. *thaliana*), followed by the NL-type. Notably, there were fewer *NBS-LRR* genes in the orchids than in the *A*. *thaliana*, which was consistent with previous studies [23]. In addition, no TNL-type genes were identified in six orchids, which proved that the TIR domain degeneration is a common phenomenon in monocots, and the TNL loss may be potentially driven by *NRG1*/*SAG101* pathway deficiency [18, 24, 25].

**Table 1:** The types and numbers of identified *NBS* genes in six orchids and *A*. *thaliana*

Species		*D. officinale*	*D. chrysotoxum*	*D. nobile*	*P. equestris*	*V. planifolia*	*A. shenzhenica*	*A. thaliana*
NBS-LRR subclass	CNL	10	14	18	7	2	4	40
	CNLCN	1	1	0	0	0	0	0
	CNNL	0	0	0	1	0	0	0
	NL	9	9	14	3	2	3	18
	NLNL	1	0	0	0	0	0	0
	NLNNL	1	0	0	0	0	0	0
	NNL	0	1	0	0	0	0	0
	^a^TNL	0	0	0	0	0	0	48
	TNLC	0	0	0	0	0	0	1
	TNLT	0	0	0	0	0	0	4
	TNNL	0	0	0	0	0	0	1
	NLT	0	0	0	0	0	0	2
	Total	22	25	32	11	4	7	114
Non-NBS-LRR subclass	CN	24	30	57	18	6	3	40
	CNC	1	0	0	0	0	0	0
	CNCN	0	0	3	1	0	0	0
	N	27	58	75	21	1	4	22
	NN	0	2	1	0	0	0	0
	NNN	0	1	0	0	0	0	0
	NC	0	0	0	6	0	0	0
	NNC	0	1	0	0	0	0	0
	TN	0	0	0	0	0	0	31
	TNN	0	0	0	0	0	0	2
	TNC	0	1	1	0	1	1	0
	RN	0	0	0	0	0	0	1
	Total	52	93	137	46	8	8	96
The total number of *NBS* genes		74	118	169	57	12	15	210

^a^ There were no TNL-type *NBS* genes identified in six orchids. The types of *NBS* genes were denoted by the abbreviation of the domains. CNL: CC-NBS-LRR; CNLCN: CC-NBS-LRR-CC-NBS; CNNL: CC-NBS-NBS-LRR; NL: NBS-LRR; NNL: NBS-NBS-LRR; NLNL: NBS-LRR-NBS-LRR; NLNNL: NBS-LRR-NBS-NBS-LRR; CN: CC-NBS; TNC: TIR-NBS-CC; RN: RPW8-NBS; CNCN: CC-NBS-CC-NBS; CNC: CC-NBS-CC; N: NBS; NN: NBS-NBS; NNN: NBS-NBS-NBS; NC: NBS-CC; NNC: NBS-NBS-CC

### Phylogenetic analysis

The 52 chloroplast (cp) genes and ITS sequences, which can well describe the phylogenetic relationships of plants [26, 27], were used to reconstruct the phylogenetic relationships of orchids (Fig. S11). Most nodes were highly supported with ML/BI bootstrap values >  = 73%/98%, except for the tree node of genus *Goodyera*, which is consistent with the fact that the cp genome variation rate was slow [26, 27]. The results showed that the phylogenetic relationships between *Vanilla* and *Dendrobium* were not close, which was by the fact that their chromosome numbers were different. In the genus *Dendrobium*, *D*. *nobile* was more closely related to *D*. *officinale*, followed by *D*. *chrysotoxum*, which was consistent with the previous study [1].

**Fig. 1:**
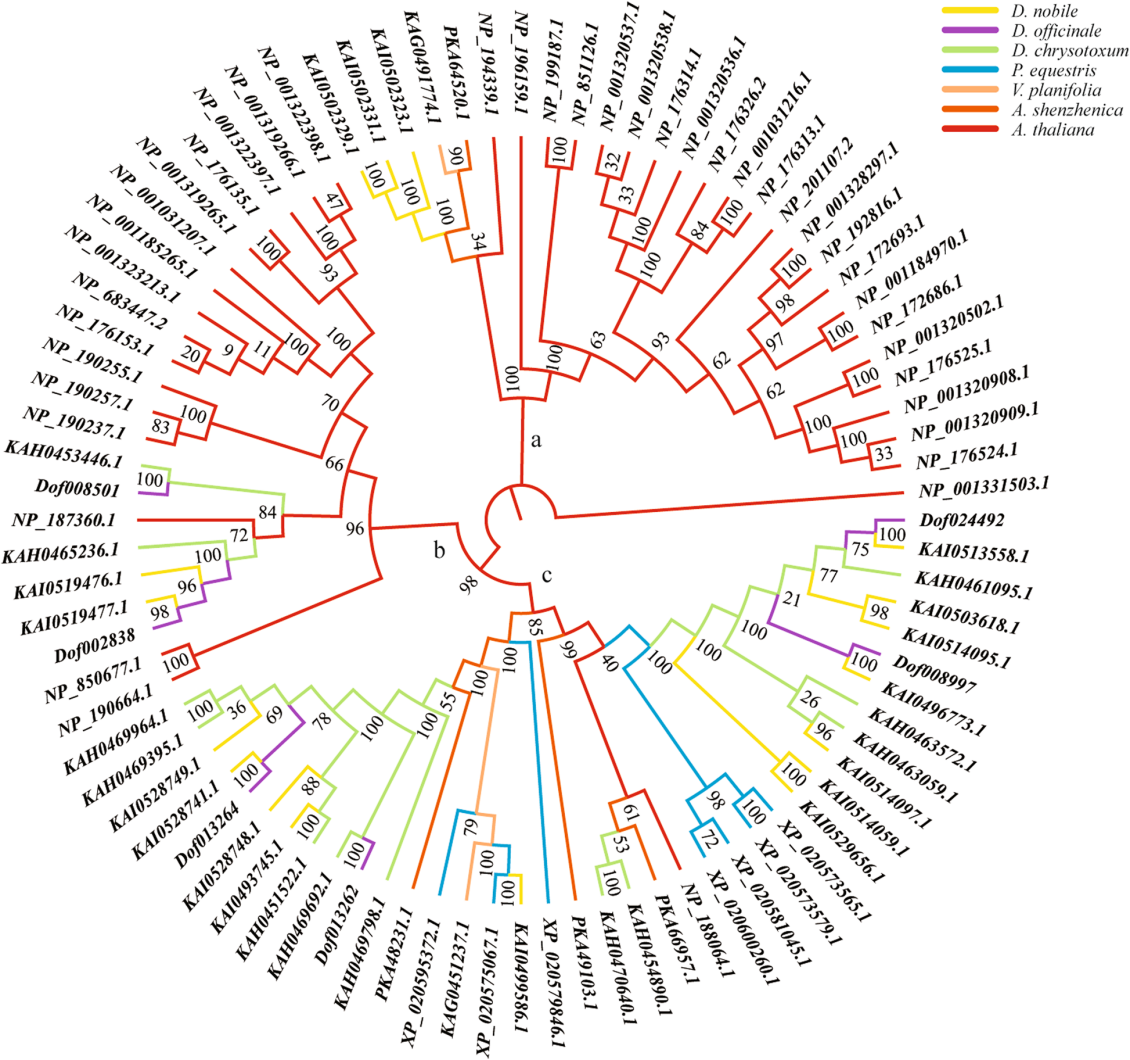
The ML phylogenetic tree of *CNL* genes. *CNL* genes are mainly divided into three branches: a, b and c. Different colors represent different species, including *D*. *officinale*, *D*. *nobile*, *D*. *chrysotoxum*, *V*. *planifolia*, *A*. *shenzhenica*, *P*. *equestris* and *A*. *thaliana*

To investigate the phylogenetic relationships of CNL-type *NBS-LRR* genes (*CNL* genes) in orchids, the ML phylogenetic trees were reconstructed using the protein sequences of 6 *D*. *officinale* genes, 17 *D*. *nobile* genes, 12 *D*. *chrysotoxum* genes, 7 *P*. *equestris* genes, 2 V. *planifolia* genes, 4 *A*. *shenzhenica* genes and 40 *A*. *thaliana* genes (Fig. 1). The results showed that the *CNL* genes were mainly divided into three branches (a, b and c) in orchids. The phylogenetic results of *CNL* genes in each branch were basically consistent with the orchid species tree (Fig. 1, Fig. S1). However, except for *A*. *thaliana* genes, there were only *D*. *nobile* and *V*. *planifolia* genes in branch a, while there were only *D*. *nobile*, *D*. *officinale* and *D*. *chrysotoxum* genes in branch b, which indicated that the orchid *CNL* genes have significantly degenerated on branches a and b. At the same time, the orchid *CNL* genes accounted for 97.4% (37/38) in branch c, which suggested that the orchid *CNL* genes have undergone significant expansions in branch c.

### Syntenic gene analysis

**Fig. 2:**
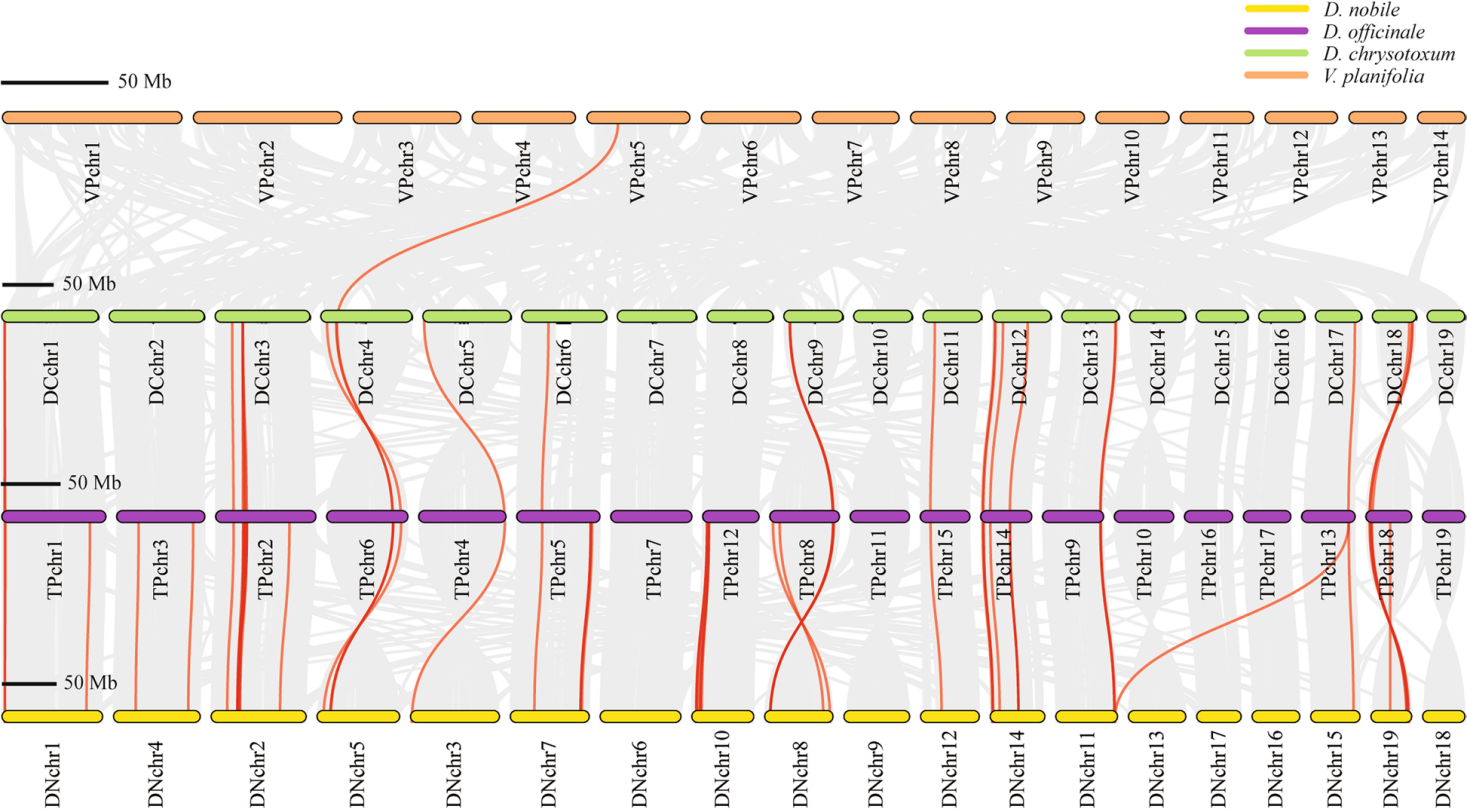
The syntenic gene analysis results among *D*. *officinale*, *D*. *nobile*, *D*. *chrysotoxum* and *V*. *planifolia*. The gray lines represent colinear gene clusters and the red lines represent orthologous *NBS* gene pairs

Synteny analysis was conducted on the *NBS* genes to investigate the gene duplication events using BLASTP and MCScanX. The results revealed that the 19 chromosomes of *D*. *chrysotoxum*, *D*. *officinale* and *D*. *nobile* were highly homologous (Fig. 2). The chromosome collinearity results of the three *Dendrobium* species showed the corresponding relationships among 19 chromosomes. However, due to the distant phylogenetic relationships, complex collinear relationships were found between the chromosomes of *Vanilla* and *Dendrobium*. Meanwhile, the highlighted red lines represented the presence of many homologous *NBS* gene pairs between the three *Dendrobium* species, which indicated orthologs may be the main way of *NBS* gene origins in *Dendrobium* species.

### *NBS* gene homology analysis

To explore the origin of the *Dendrobium NBS* genes, the results of BLASTP and MCScanX were used to sort the homologous *NBS* gene pairs between *Dendrobium* species. Based on the results of phylogenetic analysis (as shown in Fig. S1), it was assumed that there were orthologs between the *D*. *chrysotoxum* and *D*. *officinale NBS* genes, and the homologous genes pairs between *D*. *officinale* and *D*. *nobile* can be explored from the synteny analysis results. The homologies of the *NBS* genes were classified into three types (ortholog, homochromosomal duplication and heterochromosomal duplication) (Table S6). The results showed that there were 76 orthologous genes, 94 homochromosomal duplication genes and 39 heterochromosomal duplication genes in three *Dendrobium* species. The gene number differences indicated that the *NBS* gene number expansions are common events and might before the divergence of families [18, 28, 29].

**Fig. 3:**
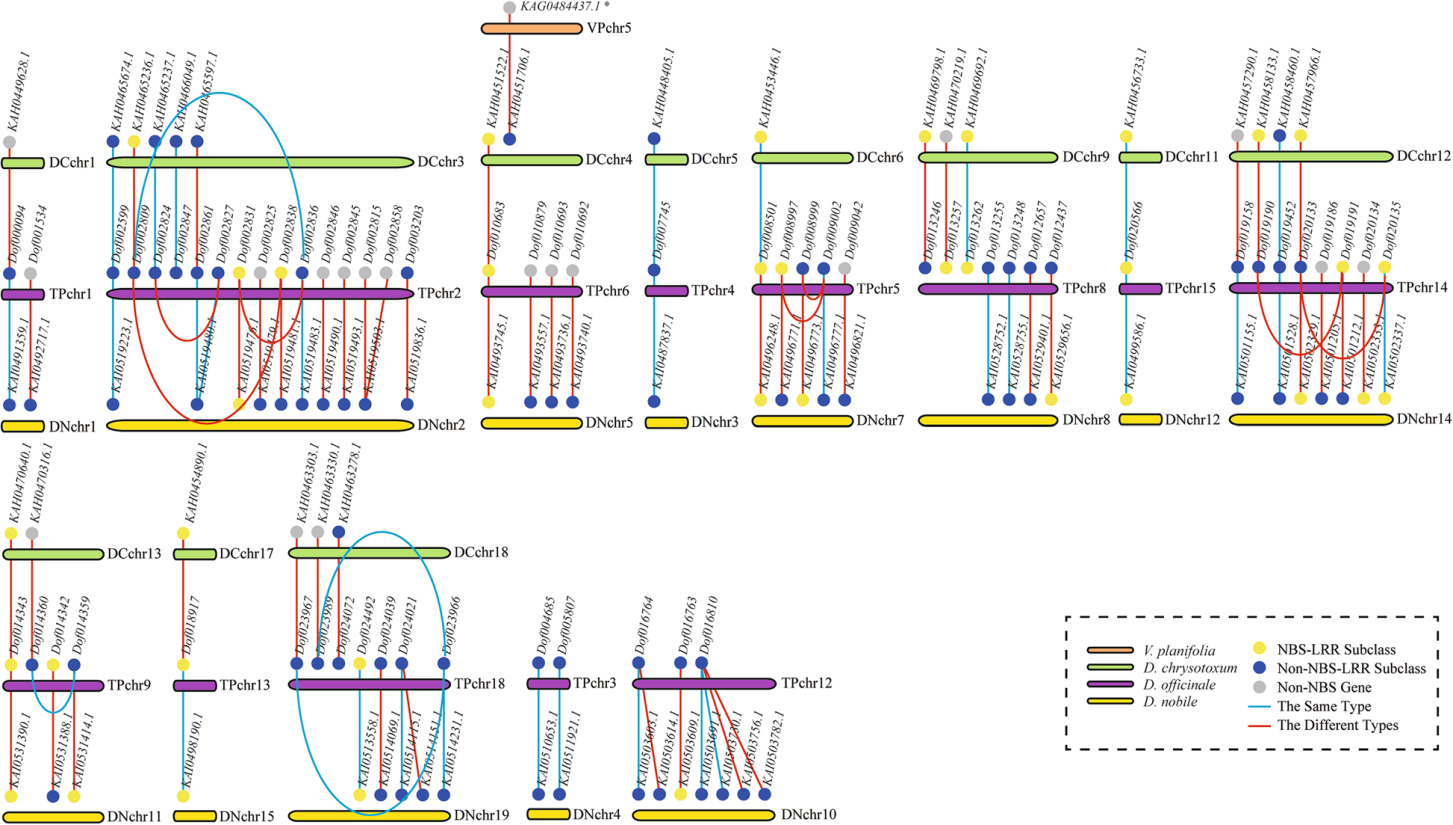
The *NBS* gene homology analysis results among *D*. *officinale*, *D*. *nobile*, *D*. *chrysotoxum* and *V*. *planifolia*. The straight lines represent the orthologous relationships between gene pairs, while curves suggest that the homology type of *NBS* genes was homochromosomal duplication or heterochromosomal duplication. Blue represents that the *NBS* gene types between gene pairs were the same, and red represents that the types between gene pairs were different. * The only gene, which was non-NBS gene, was explored from the *NBS* gene homology analysis

After arranging the different homology types of *NBS* genes, the results showed that there were at least 66 orthologous lineages, which were widely distributed in 13 chromosome lineages (Fig. 3). The blue lines represented that there were orthologous relationships between two same type *NBS* genes, while the red lines represented that the types of *NBS* genes were different. Remarkably, most of the orthologous lineages were variable, except for four lineages (*KAH0465674.1*-*Dof002599*-*KAI0519223.1*, *KAH0448405.1*-*Dof007745*-*KAI0487837.1*, *KAH0456733.1*-*Dof020566*-*KAI0499586.1*, *KAH0458460*.1-*Dof019452*-*KAI0501528.1*), which indicated that the types of most *NBS* genes had changed after originating from ortholog events.

Remarkably, the 12 extant *V*. *planifolia NBS* genes were all not the orthologous genes of the *D*. *chrysotoxum NBS* genes, except for *KAG0484437.1*, which has lost the NB-ARC domain (Fig. 3). In addition, homochromosomal duplication relationships were used to explore the *D*. *officinale NBS* gene origin. It was found that there were 40 homologous lineages incomplete, which suggested that the *NBS* gene degenerations were common phenomena in *Dendrobium*.

### Gene structure and conserved motif analyses of *NBS-LRR* genes

**Fig. 4:**
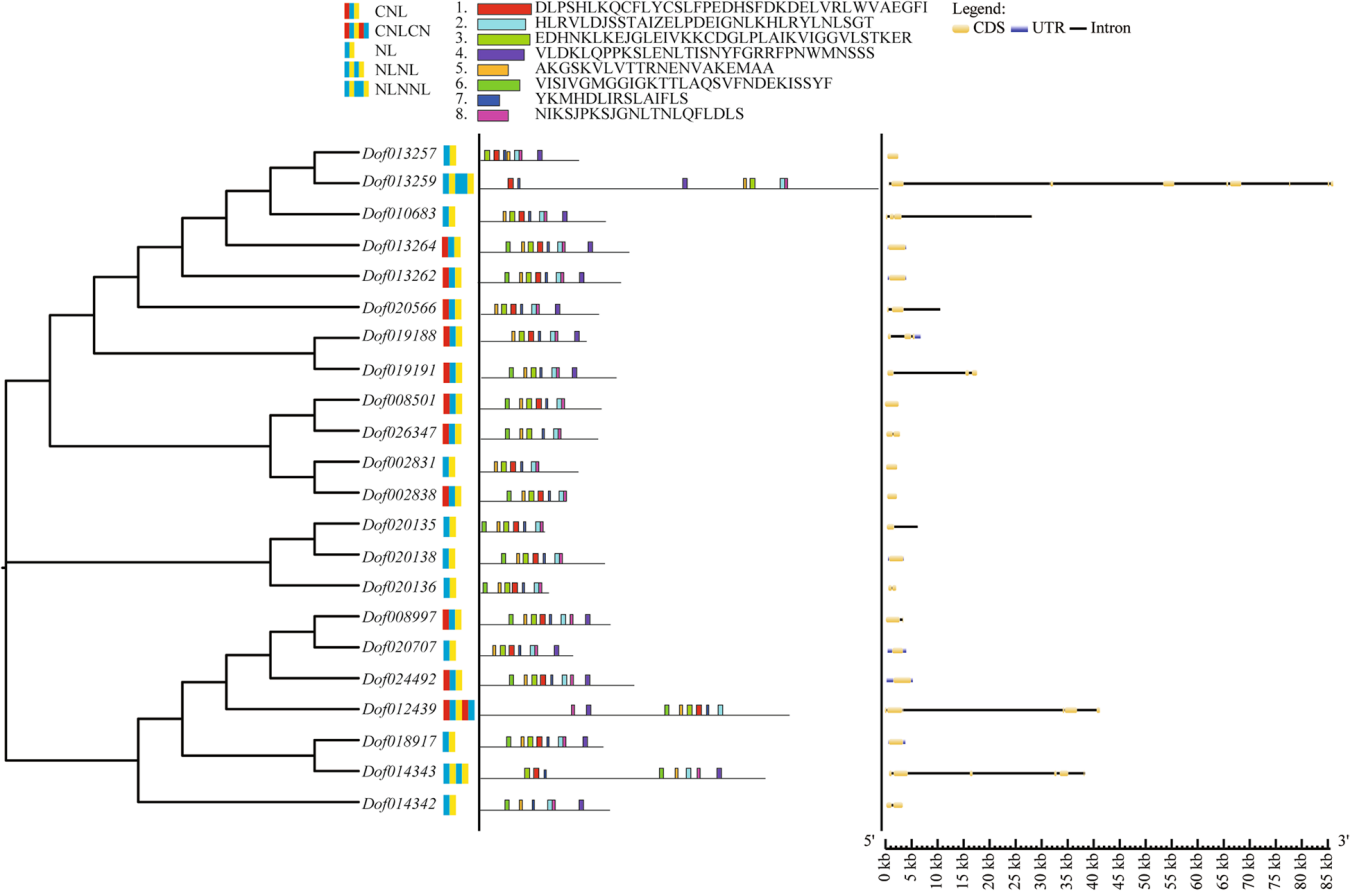
The results of phylogenetic analysis, gene structure analysis and conserved motif analysis of *D*. *officinale NBS-LRR* genes. Different color combinations represent different types of *NBS-LRR* genes

The subsequent analyses were focused on the *D*. *officinale NBS-LRR* genes, which contained the reserved NB-ARC and LRR domains. The comparison analyses of exon number, gene length and conserved motif were further performed to outline the structure features of *D*. *officinale NBS-LRR* genes. The results uncovered a significant positive correlation between exon number and gene length (Pearson's *r* = 0.9566, *P* < 0.05) (Fig. 4 and Table S7), which was consistent with previous studies [29]. For example, the ten genes (*Dof002831*, *Dof002838*, *Dof008501*, *Dof013257*, *Dof013264*, *Dof013262*, *Dof018917*, *Dof020138*, *Dof020707* and *Dof024492*) had only one exon with the lengths ranging from 1,896 bp to 5,070 bp, while *Dof013259* had 11 exons with the length of 85,500 bp.

MEME results revealed that the conserved motif orders of NL-type and CNL-type *NBS-LRR* genes were conserved (motif 6—motif 5—motif 3—motif 1—motif 7—motif 2—motif 8—motif 4), while other types (CNLCN, NLNL and NLNNL) were highly variable (Fig. 4), suggesting that NL-type and CNL-type *NBS-LRR* genes possibly bore stronger positive selection.

### Identification of *cis*-elements of *NBS-LRR* genes

**Fig. 5:**
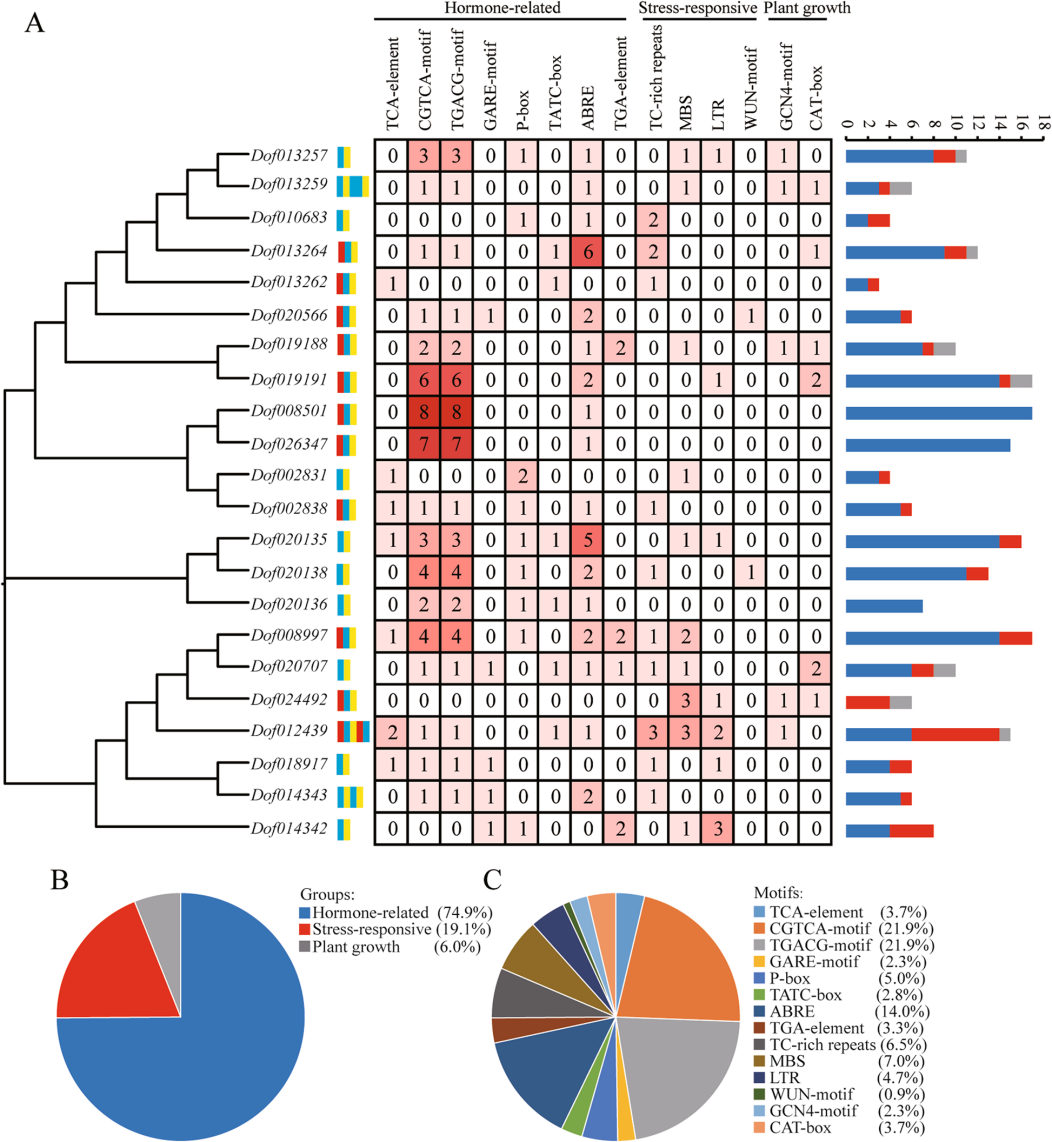
The identification results of *cis*-elements of *NBS-LRR* genes. **a** The detailed information of the types and numbers of *cis*-elements; **b** The proportion information of the numbers of *cis*-elements in different categories; **c** The proportion information of the numbers of different *cis*-elements

The 2,000 bp upstream regions of the initiation codon (ATG) were analyzed to ascertain the potential biological roles of *D*. *officinale NBS-LRR* genes using the PlantCARE tool. The *cis*-elements in the promoter regions were classified into three categories: hormone-related (74.9%), stress-responsive (19.1%) and plant growth (6.0%) (Fig. 5, Table S8). In the hormone-related category (161/215), TCA-element was involved in SA responsiveness and distributed in *Dof002831*, *Dof002838*, *Dof008997*, *Dof012439*, *Dof013262*, *Dof018917* and *Dof020135*. In the stress-responsive category (41/215), various elements related to defense and stress responsiveness (14, 6.5%), drought responsiveness (15, 7.0%), low temperature responsiveness (10, 4.7%) and wound responsiveness (2, 0.9%). Only a few *cis*-elements (13/215) were related to plant growth (Table S8). The above results revealed that there were plenty of hormone-related and stress-responsive *cis*-elements in the promoter regions of *D*. *officinale NBS-LRR* genes, which were consistent with *Asparagus officinalis NBS-LRR* genes [30].

### Functional annotation of *D*. *officinale NBS-LRR* genes

The 22 *D*. *officinale NBS-LRR* genes were annotated with GO and KEGG databases to explore the role of *NBS-LRR* genes in *D*. *officinale*. All genes had the molecular function of ADP binding, which was due to the conserved structure of the NB-ARC domain (Table S9). Eight genes (*Dof002831*, *Dof002838*, *Dof008501*, *Dof026347*, *Dof019188*, *Dof020135*, *Dof020136* and *Dof020138*) could be playing a role in the plant-pathogen interaction pathway, which belongs to the ETI system and responds to bacterial effectors. The results revealed that CNL-type and NL-type *NBS-LRR* genes were widely distributed in the *D*. *officinale* ETI system. Moreover, six genes (*Dof010683*, *Dof013257*, *Dof013259*, *Dof013262*, *Dof013264* and *Dof020566*) may regulate gene expression by taking part in the Ras signaling pathway. All results indicated that *NBS-LRR* genes participated in the *D*. *officinale* immune systems upstream and downstream.

### *NBS-LRR* gene expression profiles in response to SA

**Fig. 6:**
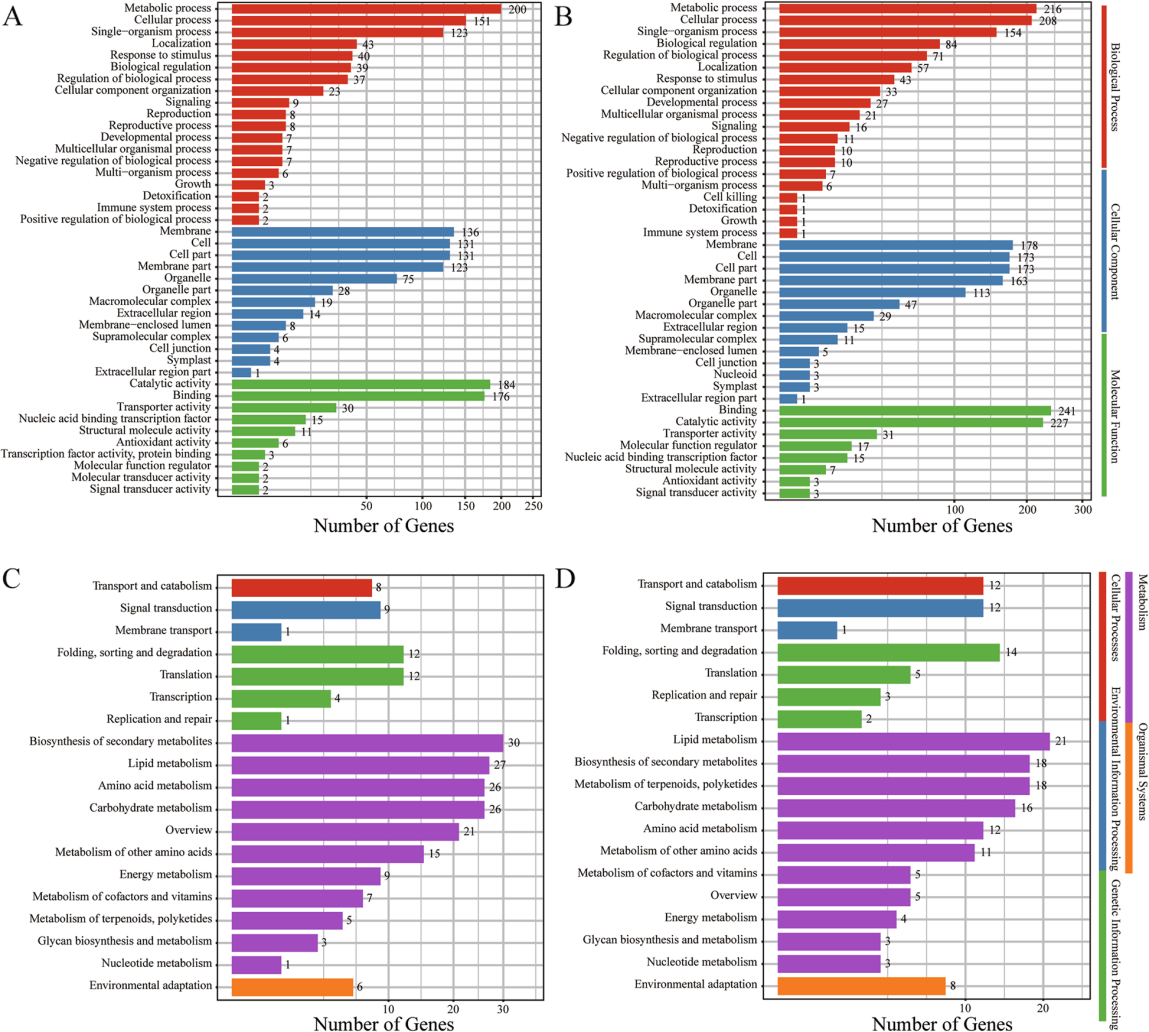
The GO and KEGG annotation results of DEGs. **a** The GO classification results of up-regulated DEGs; **b** The GO classification results of down-regulated DEGs; **c** The KEGG classification results of up-regulated DEGs; **d** The KEGG classification results of down-regulated DEGs

**Fig. 7:**
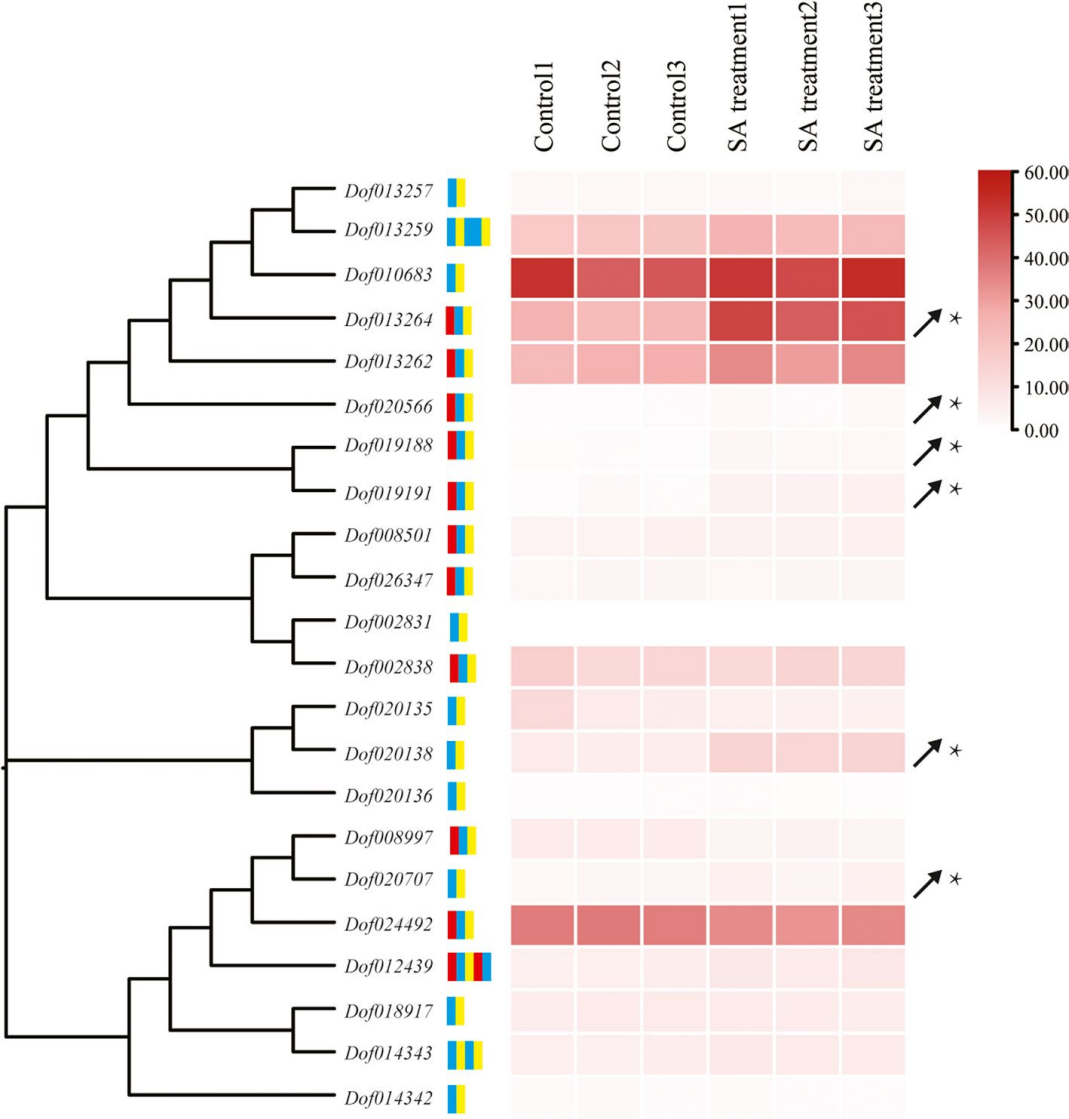
The expression heat map of *D*. *officinale NBS-LRR* genes by SA treatment. The symbol * represents the significant up-regulation (foldchange > 1.5 ×)

SA can regulate the expression levels of *R* genes to activate the resistance response to biotic stress [12]. To evaluate whether *D*. *officinale NBS-LRR* genes were in response to SA treatment, *NBS-LRR* gene expression patterns were investigated. From the transcriptome sequencing, a total of 145,498,271 clean reads were obtained, and all of the Q30 base percentages were above 94.4% (Table S10A; BioProject accession: PRJNA851113). In addition, the clean reads mapped to the *D*. *officinale* reference genome ranged from 90.96% to 91.57% (Table S10B), and 1,677 DEGs were identified (Table S10C). The up-regulated and down-regulated DEGs were evenly annotated to biological processes, cellular components and molecular functions (Fig. 6A and B), while the DEGs mainly belonged to the metabolism pathways, including biosynthesis of other secondary metabolites, lipid metabolism, amino acid metabolism, carbohydrate metabolism, metabolism of other amino acids, energy metabolism, metabolism of cofactors and vitamins, metabolism of terpenoids and polyketides, glycan biosynthesis and metabolism and nucleotide metabolism (Fig. 6C and D). The relative expression levels were represented by FPKM values, which were calculated with transcriptome data. The results showed that the expression levels of six *NBS-LRR* genes (*Dof013264*, *Dof020566*, *Dof019188*, *Dof019191*, *Dof020138* and *Dof020707*) were significantly up-regulated (foldchange > 1.5 ×) (Fig. 7). *Dof020138* and *Dof019188* both belonged to the plant-pathogen interaction pathway (Table S9), while there were no TCA-elements (salicylic acid responsiveness) found in the promoter regions (Table S8), which suggested that *Dof020138* and *Dof019188* may be indirectly up-regulated by SA in the *D*. *officinale* ETI system.

### WGCNA of *D*. *officinale* genes by SA treatment

**Fig. 8:**
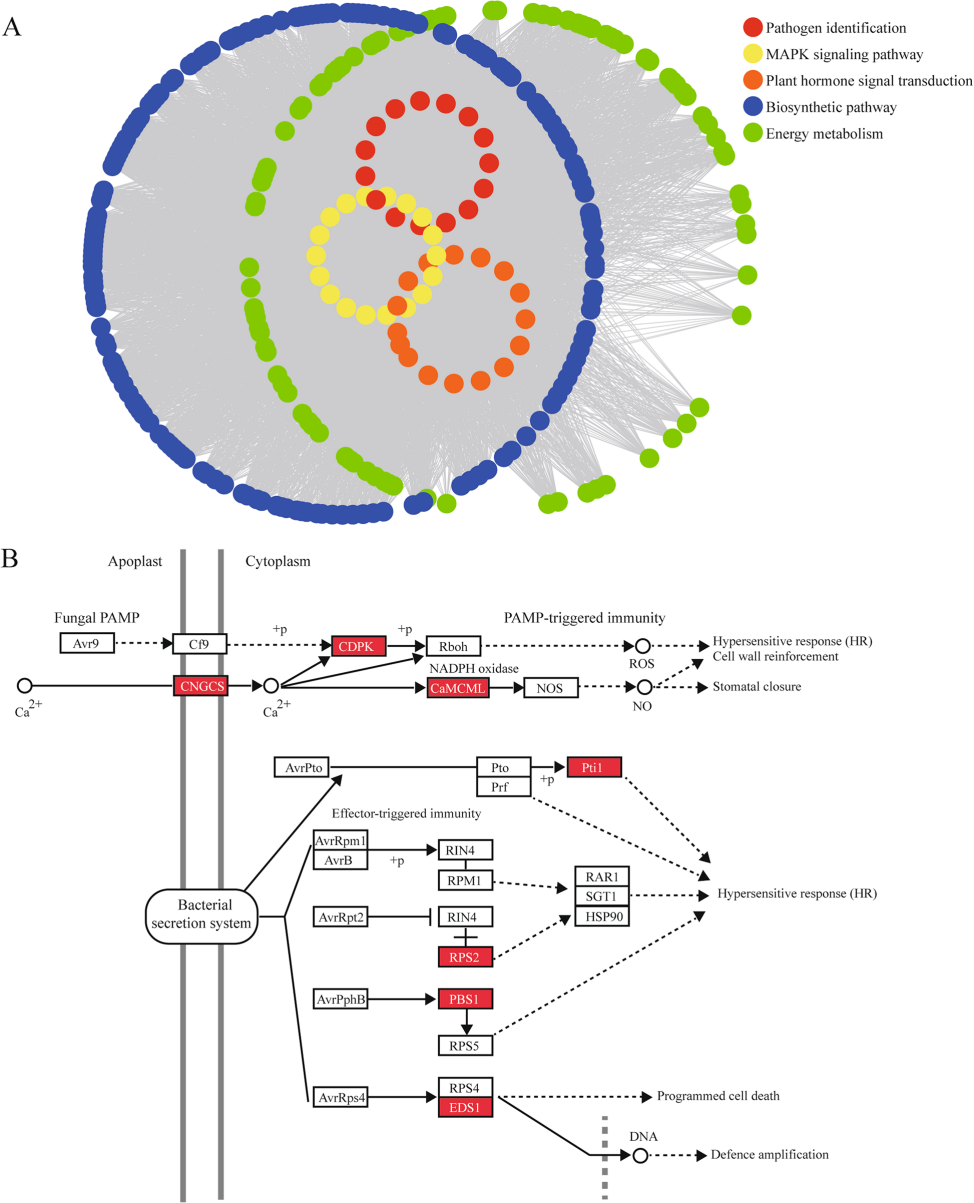
The transcriptome analysis results of *D*. *officinale* by SA treatment. **a** The network map of the significantly up-regulated turquoise module in WGCNA; **b** The schematic diagram of the PTI and ETI systems. Red blocks represent the putative genes involved in PTI and ETI system in the turquoise module

Weighted gene co-expression network analysis (WGCNA) was performed with transcriptome data to explore the immune response network of *D*. *officinale*. The results showed that the turquoise module containing the *D*. *officinale NBS-LRR* gene *Dof020138* was most positively correlated to SA treatment (Fig. S2, Table S11B). The genes in the turquoise module were classified into five categories: pathogen identification, plant hormone signal transduction, biosynthetic pathway, energy metabolism and MAPK signaling pathway (Fig. 8A, Table S11A). Remarkably, there were 15 genes belonging to the pathogen identification category, among which 11 genes belonged to the PTI system and four genes belonged to the ETI system (Fig. 8B, Table S11). The quantitative PCR results proved that the expression levels of nine genes, except for *Dof013547*, *Dof005640*, *Dof015798*, *Dof017381*, *Dof004597* and *Dof017452*, were significantly up-regulated by SA treatment (Table S12). These nine genes are widely distributed in PTI (CNGCs, CDPK and CaMCML) and ETI (EDS1 and RPS2) systems, which suggested that the *D*. *officinale* PTI and ETI systems, will be activated by the plant hormone, salicylic acid [31].

## Discussion

### *NBS* genes are highly variable in *Dendrobium*

*NBS* genes originated before the last common ancestor of green plants [19]. Nearly all *NBS-LRR* genes, of which the proteins contain both NB-ARC and LRR domains, with known functions, are involved in plant immunity [32]. The *NBS-LRR* genes are divided into three subfamilies: TIR-NBS-LRR (TNL), CC-NBS-LRR (CNL) and RPW8-NBS-LRR (RNL) [19, 33], and the divergence of them should at least predate the divergence of chlorophytes and streptophytes [19]. TNL and CNL proteins are mainly responsible for recognizing specific pathogens, while RNL proteins may play an auxiliary role in downstream defense signal transduction [25, 29]. The NB-ARC domain can bind ATP/GTP, resulting in phosphorylation to transmit disease resistance signals downstream [34]. The LRR domain is typically involved in protein–protein interactions, which generally takes the role of pathogen recognition [35]. Consequently, the sequences of the NB-ARC domain encoded by different *NBS-LRR* genes are highly conserved, while the LRR domain is highly variable [36].

*NBS* genes not only expand greatly in plant genomes [25] but also degenerate rapidly [30, 37], which leads that the numbers of *NBS* genes varying greatly among different species. For example, there are over 2,000 *NBS* genes in the extremely large wheat genome, but these genes are extremely scarce in orchids, which is in accord with our results [18, 23, 38]. As a result, plants have a wide variety of *NBS* genes that can identify more pathogens and thus improve their ability to induce defense responses [39].

In this study, phylogenetic analysis, syntenic gene analysis and gene homology analysis were performed to speculate on the evolution of *Dendrobium NBS* genes. All results showed that *Dendrobium NBS* genes are highly variable in structures. On the one hand, the types of homologous *NBS* genes in *Dendrobium* are changing generally. For example, the 62 of 66 homologous lineages, which widely distributed in 13 chromosome lineages, cannot remain the same type throughout the lineages (Fig. 3). On the other hand, A large number of *NBS* genes are degenerating, which refers to the loss of the NB-ARC domain. Firstly, *CNL* genes degenerated in branches a and b (Fig. 1). Secondly, among 66 homologous lineages, 40 homologous lineages were not complete (Fig. 3), which indicated that most *NBS* genes were degenerating. It is assumed that large numbers of *NBS* gene expansions from orthologs and duplication events are the basic premise of *NBS* diversity, which adapted plants to identify more pathogens [24, 39]. In conclusion, *NBS* genes are highly variable in the genus *Dendrobium*.

### *NBS-LRR* genes play important roles in *D*. *officinale* immune systems and signal transduction pathways

*D*. *officinale* is a valuable TCM and is known as “The first of China’s nine immortal herbs” [5]. With the development of *D*. *officinale* industrialization, it is urgent to improve the resistance of tissue culture seedlings by genetic engineering. It is common for *D*. *officinale* to be exposed to various pathogens, including fungus, bacteria, and viruses during industrial cultivation [6–9]. For example, the common and destructive fungal pathogen, *F*. *oxysporum*, always causes stem rot of *D*. *officinale* and has a high incidence of 30% to 50% [8, 40]. The medicinal part of *D*. *officinale* is the stem segment, so pathogen infection in stem segments can lead to huge economic losses.

To explore the roles of *NBS-LRR* genes in *D*. *officinale*, structure feature, *cis*-elements and functional annotation analyses were performed on the *D*. *officinale NBS-LRR* genes. All results suggested that *D*. *officinale NBS-LRR* genes were homologous to proteins in immune systems and signal transduction pathways. In the plant hormone signal transduction map, *Dof008997* and *Dof024492* were annotated as DELLA proteins, which promote stem growth and induce germination in the gibberellin signaling pathway (Table S9). In the Ras signaling pathway, *Dof010683*, *Dof013257*, *Dof013259*, *Dof013262*, *Dof013264* and *Dof020566* were all annotated as SHOC2 proteins, which regulate the MAPK signaling pathway upstream (Table S9). Remarkably, there were two *R* genes, *RPM1* (*Dof002831*, *Dof002838*, *Dof008501*, *Dof026347* and *Dof019188*) and *RPS2* (*Dof020135*, *Dof020136* and *Dof020138*), annotated in the *D*. *officinale* ETI system, both of which respond to bacterial effectors to activate hypersensitive response (Table S9). In conclusion, most *NBS-LRR* genes (16/22) may play roles in *Dendrobium* immune systems and signal transduction pathways.

### *Dof020138* is indirectly activated by SA in the *D*. *officinale* ETI system

The plant immune systems are activated by signaling transduction networks, such as calcium (Ca^2+^), reactive oxygen species (ROS) and hormones [31, 41]. In addition to Ca^2+^ and ROS, plant hormones, such as SA, JA and ABA, could be the primary signaling molecules that function in the regulation of plant immunity [42]. SA signaling, which might have originated in the last common ancestor of land plants [12, 43], participates in the resistance response to biotrophic pathogens by regulating the expression levels of *R* genes [44, 45]. In previous studies, *NBS-LRR* genes that can be up-regulated by SA have been found in many species, such as *Arachis hypogaea* (*AhRRS5*, *AhRAF4*) [46, 47], *Gossypium hirsutum* (*GbaNA1*) [48], *Zea mays* (*ZmNBS25*) [49], *Triticum aestivum* (*TaRPM1*) [50], *Manihot esculenta* (*MeLRR*s) [51] and *Glycine max* (*SRC7*) [52]. To explore the molecular basis of disease resistance in *D*. *officinale*, healthy one-year seedlings were treated with SA. The results of transcriptome data and qPCR showed that the expression levels of several genes in the PTI and ETI systems were up-regulated, and a large number of genes related to biomolecule synthesis and energy metabolism were mobilized in plant cells. Remarkably, *Dof020138* (*RPS2*) and *Dof019188* (*RPM1*) may be indirectly up-regulated by SA in the ETI system (Table S9, Table S12), but only *Dof020138* were uncovered in the turquoise module from the WGCNA results.

The expression level of RPS2 protein, which receives signals from the effector protein AvrRpt2 by being antagonized to RIN4 protein, was significantly up-regulated in the ETI system [53]. Afterward, the protein of *Dof020138*, which was suppressed by RIN4 protein, triggers a hypersensitive response by transducing signals to downstream proteins, such as RAR1, SGT1 and HSP90 (Table S9, Fig. 8B) [54–57]. However, whether Dof020138 protein performs the function of RPS2 protein and the detailed mechanism of *Dof020138* regulated by SA needs further study. At the same time, *Dof020138* has comprehensive associations with other genes in the pathogen identification pathways, MAPK signaling pathways, plant hormone signal transduction pathways, biosynthetic pathways and energy metabolism pathways, which suggests that *Dof020138* may perform a non-negligible function in the overall mobilization of the *D*. *officinale* immune system by SA. In conclusion, the SA can indirectly activate the *D*. *officinale NBS-LRR* gene *Dof020138* in the ETI system.

## Conclusions

In this study, the genomes of six orchids and *A*. *thaliana* were used to identify the *NBS* genes, and the CNL-type NBS proteins were used to reconstruct ML phylogenetic trees. We found that the *NBS* genes in Orchidaceae species were degenerating generally. The *Dendrobium NBS* gene homology analysis showed that the *Dendrobium NBS* genes were diversified. The *D*. *officinale NBS-LRR* genes were used for gene structure and conserved motif analyses, *cis*-elements analysis and functional annotation analysis, which revealed that *NBS-LRR* genes take parts in the ETI system, plant hormone signal transduction pathway and Ras signaling pathway. In addition, SA treatment transcriptome data was used for exploring the molecular basis of *D*. *officinale* immune systems. All results indicated that *Dendrobium NBS* genes are highly variable during long-term expansion and degeneration events, and the *D*. *officinale NBS-LRR* gene *Dof020138*, which may have important breeding value, is indirectly activated by SA in the ETI system.

## Methods

### Identification of *NBS* genes in Orchidaceae

The newest genome data of *Dendrobium officinale* Kimura et Migo (ID: 31,795), *Dendrobium nobile* Lindl. (ID: 17,836), *Dendrobium chrysotoxum* Lindl. (ID: 41,833), *Vanilla planifolia* Andrews (ID: 17,745), *Apostasia shenzhenica* Z.J.Liu & L.J.Chen (ID: 66,931), *Phalaenopsis equestris* (Schauer) Rchb. (ID: 11,403) and *Arabidopsis thaliana* (L.) Heynh. (ID: 4) were downloaded from the public databases (NCBI). Two strategies, HMM and BLAST searches [58, 59], were performed to identify *NBS* genes in these six orchids and *A*. *thaliana*. Firstly, the protein sequences were mapped and trained against the model of the NB-ARC (PF00931), Toll-Interleukin receptor (TIR, PF01582), Leucine-rich repeat (LRR, PF00560, PF07723, PF07725, PF12799, PF13306, PF08191 and PF13855) and RPW8 (PF05659) domains using hmmer3.0 with default parameters (Table S1A). Secondly, the reference protein sequences were downloaded from the NCBI protein database to contain as many known *NBS* genes as possible by searching GeneBank with the keywords: “*Arabidopsis* NB-ARC”, “*Arabidopsis* LRR”, “*Arabidopsis* TIR” and “*Arabidopsis* RPW8”. The 121 sequences of the *Arabidopsis* proteins with typical features of *NBS* genes were treated as seed sequences (Table S1B) and aligned as queries to the corresponding genome using BLASTP (Table S1C).

The HMM and BLASTP results were filtered and classified by the Conserved Domain website (https://www.ncbi.nlm.nih.gov/Structure/cdd/wrpsb.cgi) (Table S1D) [60], the Pfam database (http://pfam.xfam.org/) (Table S1E) [61] and the SMART website (http://smart.embl-heidelberg.de/) [62]. The genes that contained significant NB-ARC domains were retained as the putative *NBS* genes. For the identification of coil-coiled (CC) motifs, the DeepCoil2 program (https://toolkit.tuebingen.mpg.de/tools/deepcoil2) was performed with a threshold value of 0.5 [63]. The types of *NBS* genes were determined according to the orders of NB-ARC (N), TIR (T), CC (C), LRR (L) and RPW8 (R) domains.

### Sequence alignment and phylogenetic analysis

The 52 cp genes and ITS sequences of 25 Orchidaceae species, two Araceae species and *A*. *thaliana* were used to reconstruct the Maximum Likelihood (ML) and BI phylogenetic trees (Table S2). The sequences were aligned using MAFFT 7.220 [64]. Under the rule of the Akaike Information Criterion (AIC), the optimum base substitution model calculated by Modeltest 3.7 was GTR + I + Γ [65]. The ML phylogenetic tree was constructed using RAxML 7.4.2 with 1,000 rapid bootstrap inferences [66], and the outgroup was *A*. *thaliana*. The BI analysis was made using MrBayes 3.2.7 with 1,000,000 generations [67]. Trees were sampled every 1,000 generations, and the first 25% of these were discarded. The remaining trees were used to build the Bayesian tree of posterior probabilities.

The alignments of CNL-type NBS protein sequences were performed using ClustalX2.1 with the complete alignment [68]. After removing the seven genes (*KAH0456733.1*, *Dof019191*, *KAH0457269.1*, *Dof026347*, *Dof019188*, *Dof020566* and *KAI0514091.1*), which lacked the conserved regions, 88 *CNL* genes were used to reconstruct phylogenetic trees. The phylogenetic trees were estimated using MEGA X by the ML method with the following parameters: Poisson model, pairwise deletion and 1,000 bootstrap replicates [69].

### Gene duplication analysis

The MCScanX software was performed to search for gene duplication events between four chromosome-level genomes (*D*. *officinale*, *D*. *nobile*, *D*. *chrysotoxum* and *V*. *planifolia*) [70]. All the protein sequences were compared using all-vs-all BLASTP with parameters: V = 10, B = 100, filter = seg, E-value < 1e-10, and the output format was set as tabular format (-m 8). The resulting blast hits were incorporated along with chromosome coordinates as input for MCScanX analysis. The chromosomes were renamed according to the chromosome lengths (Table S3).

### Prediction of homologous genes

For the prediction of *Dendrobium NBS* gene origins, the MCScanX results were used to determine the orthologous genes first. The paralogous genes of other *NBS* genes were conjectured by the BLASTP results (homochromosomal duplication and heterochromosomal duplication).

### Gene structure and conserved motif analyses in *D*. *officinale*

The CDS information was shown to investigate the structural variations of *D*. *officinale NBS-LRR* genes using the online program Gene Structure Display Server (http://gsds.gao-lab.org/) [71]. The protein sequences of 22 *NBS-LRR* genes were submitted to the motif analysis using the online tool MEME Suite (https://meme-suite.org/meme/) [72] with the following settings: (1) optimum motif width was set to 6 and 50; (2) number of motifs was eight with an E-value < 1e-10.

### *Cis*-elements analysis

The promoter sequences (2,000 bp upstream of the translational start site) of *D*. *officinale NBS-LRR* genes were obtained. Afterward, the online software PlantCARE (http://bioinformatics.psb.ugent.be/webtools/plantcare/html/) [73] was employed to investigate putative *cis*-elements in the promoter regions.

### Functional annotation of *NBS-LRR* genes

The *D*. *officinale NBS-LRR* genes were functionally annotated based on the publicly available databases including GO and KEGG databases with default parameters [74–76].

### Plant treatment, RNA extraction and sequencing

*D*. *officinale* one-year cultivated seedlings (voucher specimen: Yang202201) without obvious disease infection were selected for SA treatment with 1 mM SA, and SA-free individuals were used as the control. The treatment group and control group were set with three independent replicates, respectively. The leaves from SA treatment and the control were collected 7 d after treatment. All samples were frozen immediately in liquid nitrogen and stored at -80 ℃ until use.

The total RNA was extracted using MiniBEST Plant RNA Extraction Kit (Takara). RNA sequencing was performed using a high-throughput sequencing platform, Illumina HiSeq2500. The clean reads obtained from RNA-Seq were mapped to the *D. officinale* genome and assembled using Hisat2 and Stringtie, respectively. The differential expression genes (DEGs) were identified using the DEseq2 package in R with the standard of the adjusted *p*-value of 0.05 and the foldchange more than 1.5 × [77].

### Weighted gene co-expression network analysis

Weighted gene co-expression network analysis (WGCNA) was performed for gene co-expression network construction based on the transcriptome data. It is assumed that genes that have related functions may have similar expression profiles [78]. For the gene network, the parameters for dynamic tree cutting were as follows: maxBlockSize: 2000, minModuleSize: 30, deepSplit: 2. The network map of co-expressed genes was drawn based on the software Cytoscape [79]. The position of NBS-LRR proteins in the Plant-pathogen interaction pathway was displayed using map04626 of KEGG database [74–76].

### Quantitative real-time PCR

Quantitative real-time PCR (qPCR) was used to measure the expression levels of 15 genes (*Dof008571*, *Dof024904*, *Dof000577*, *Dof010081*, *Dof010899*, *Dof013547*, *Dof005640*, *Dof006104*, *Dof014321*, *Dof015798*, *Dof017381*, *Dof004597*, *Dof017452*, *Dof018039* and *Dof020138*), which belonged to *D*. *officinale* PTI and ETI systems. The treatment concentration and treatment time of SA were the same as above. The PrimeScript II 1st Strand cDNA Synthesis Kit (TaKaRa) was used for reverse transcription of the extracted total RNA, and LightCycler 96 real-time fluorescent quantitative PCR instrument was used for quantitative analysis. The total volume of each reaction was 20 μL, including SYBR Green I 10 μL, each primer 0.4 μL, cDNA 2 μL and ddH2O 7.2 μL. Temperature Cycles were set to default and three replicates per sample. The gene *GAPDH* was used as the internal reference gene. Primer sequences are presented in Table S4.

## Supplementary Information

**Additional file 1.**

**Additional file 2.**

**Additional file 3.**

**Additional file 4.**

**Additional file 5.**

**Additional file 6.**

**Additional file 7.**

**Additional file 8.**

**Additional file 9.**

**Additional file 10.**

**Additional file 11.**

**Additional file 12.**

**Additional file 13.**

**Additional file 14.**

## Data Availability

all of the raw data used in this study have been deposited in NCBI (BioProject accession: PRJNA851113, website: https://www.ncbi.nlm.nih.gov/bioproject/PRJNA851113). The voucher specimen (Yang202201) was identified by D.X.Y. and stored in the Institute of Plant Resources and Environment, College of Life Sciences, Nanjing Normal University.

## Abbreviations

TCM: Traditional Chinese Medicine
*R* genes: Disease resistance genes
SA: Salicylic acid
PTI: Pathogen-associated molecular patterns triggered immunity
ETI: Effector-triggered immunity
NB-ARC domain: Nucleotide binding sites domain; LRR domain: C-terminal leucine-rich repeats domain; cp: chloroplast
WGCNA: Weighted gene co-expression network analysis
ROS: Reactive oxygen species
CC: Coil-coiled
ML: Maximum Likelihood
AIC: Akaike Information Criterion
DEGs: Differentially expressed genes
qPCR: Quantitative real-time PCR
*CNL* genes: CNL-type *NBS-LRR* genes
