# Supplementary material for: Evolution patterns of NBS genes in the genus Dendrobium and NBS-LRR gene expression in D. officinale by salicylic acid treatment
Source: BMC Plant Biol. 2022 Nov 14;22:529. doi: 10.1186/s12870-022-03904-2 (PMC9661794; doi:10.1186/s12870-022-03904-2)
Supplement: Supplementary file 1 — Additional file 1. [file 12870_2022_3904_MOESM1_ESM.docx]

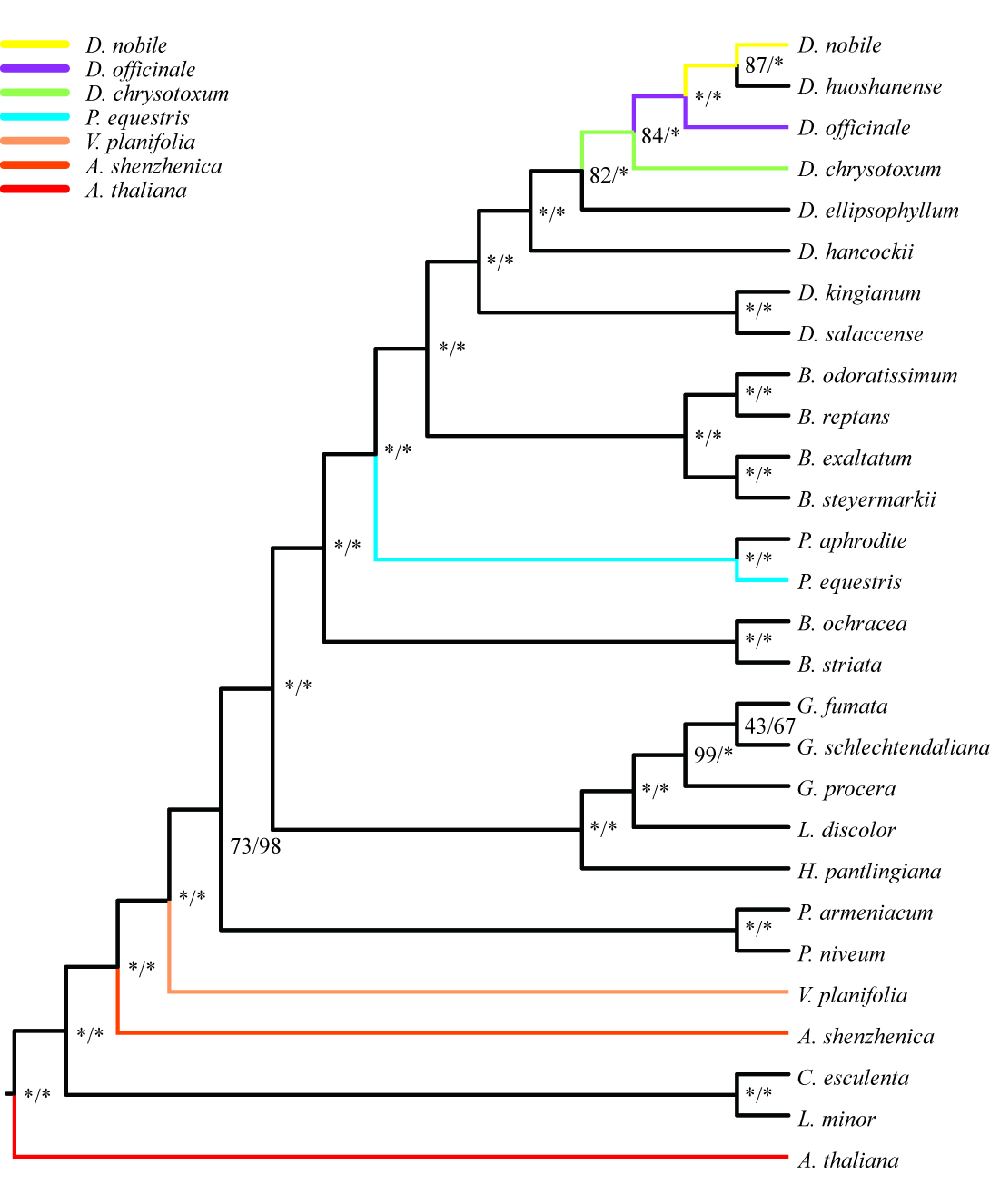


**Fig. S1** The phylogenetic tree reconstructed by cp genes and ITS sequences in orchids. The first value represents the ML bootstrap value (%), and the second represents the BI bootstrap value (%). The symbol * represents the bootstrap value of 100%
