# Supplementary material for: Evolution patterns of NBS genes in the genus Dendrobium and NBS-LRR gene expression in D. officinale by salicylic acid treatment
Source: BMC Plant Biol. 2022 Nov 14;22:529. doi: 10.1186/s12870-022-03904-2 (PMC9661794; doi:10.1186/s12870-022-03904-2)
Supplement: Supplementary file 2 — Additional file 2. [file 12870_2022_3904_MOESM2_ESM.docx]

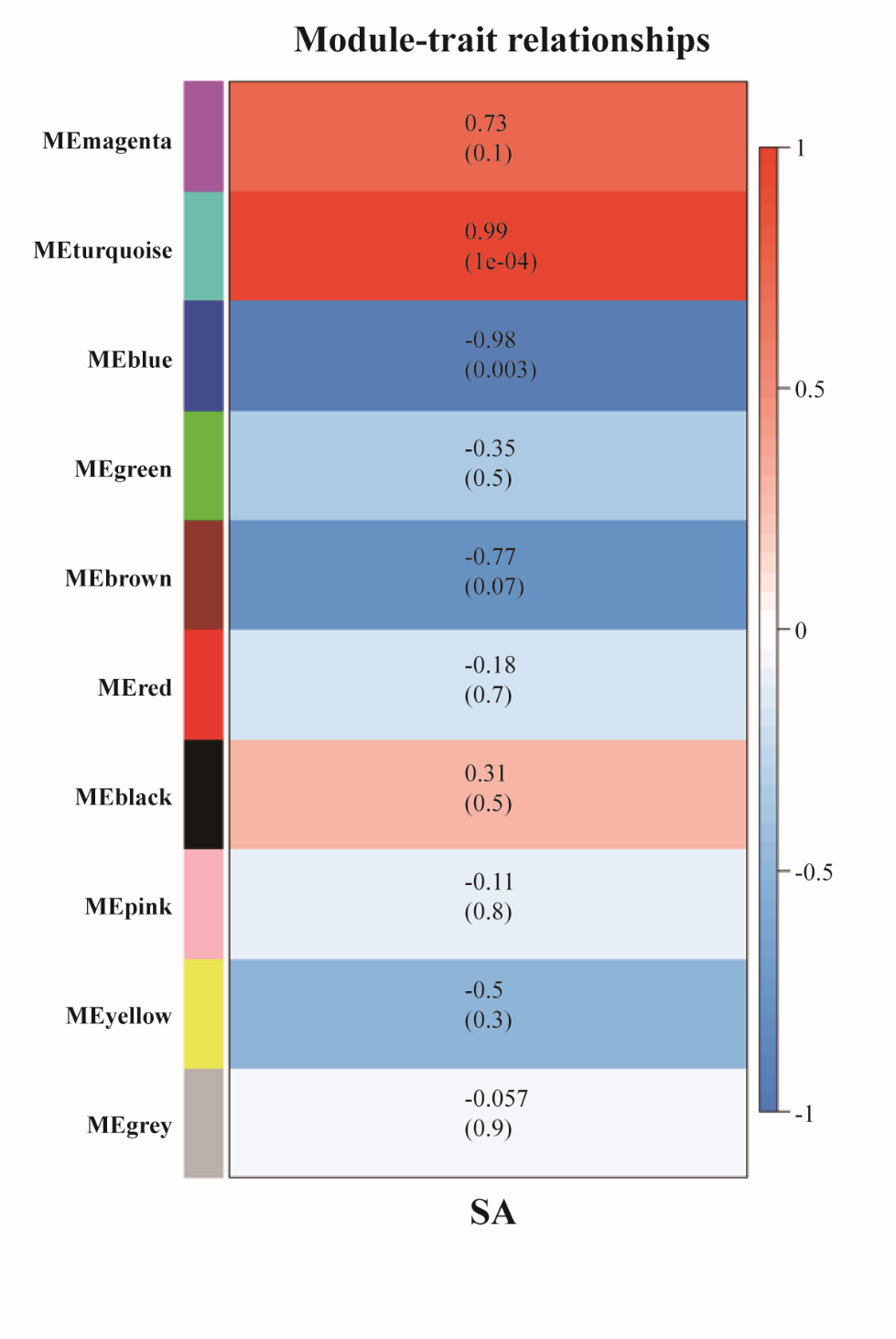


**Fig. S2** The correlation heat map between the modules and the traits in WGCNA. The red represents the positive correlation, and the blue represents the negative correlation
