# Supplementary material for: Evolution patterns of NBS genes in the genus Dendrobium and NBS-LRR gene expression in D. officinale by salicylic acid treatment
Source: BMC Plant Biol. 2022 Nov 14;22:529. doi: 10.1186/s12870-022-03904-2 (PMC9661794; doi:10.1186/s12870-022-03904-2)
Supplement: Supplementary file 9 — Additional file 9. [file 12870_2022_3904_MOESM9_ESM.docx]

| **Table S7** Gene structure information of *NBS-LRR* genes in *D. officinale* | | | | |
| --- | --- | --- | --- | --- |
| Number | Gene name | Locus | Gene length (bp) | Exon number |
| 1 | *Dof002831* | TPchr2 | 2,154 | 1 |
| 2 | *Dof002838* | TPchr2 | 1,896 | 1 |
| 3 | *Dof008501* | TPchr5 | 2,658 | 1 |
| 4 | *Dof013257* | TPchr8 | 2,160 | 1 |
| 5 | *Dof013264* | TPchr8 | 3,593 | 1 |
| 6 | *Dof013262* | TPchr8 | 3,579 | 1 |
| 7 | *Dof018917* | TPchr13 | 3,297 | 1 |
| 8 | *Dof020138* | TPchr14 | 3,072 | 1 |
| 9 | *Dof020707* | TPchr15 | 3,636 | 1 |
| 10 | *Dof024492* | TPchr18 | 5,070 | 1 |
| 11 | *Dof020135* | TPchr14 | 5,921 | 2 |
| 12 | *Dof020136* | TPchr14 | 1,546 | 2 |
| 13 | *Dof008997* | TPchr5 | 3,366 | 2 |
| 14 | *Dof014342* | TPchr9 | 3,162 | 2 |
| 15 | *Dof026347* | unanchor1602 | 2,659 | 2 |
| 16 | *Dof019188* | TPchr14 | 6,345 | 3 |
| 17 | *Dof019191* | TPchr14 | 17,289 | 3 |
| 18 | *Dof020566* | TPchr15 | 10,190 | 3 |
| 19 | *Dof010683* | TPchr6 | 28,080 | 6 |
| 20 | *Dof012439* | TPchr8 | 41,358 | 6 |
| 21 | *Dof014343* | TPchr9 | 37,730 | 8 |
| 22 | *Dof013259* | TPchr8 | 85,500 | 11 |
