# Supplementary material for: Evolution patterns of NBS genes in the genus Dendrobium and NBS-LRR gene expression in D. officinale by salicylic acid treatment
Source: BMC Plant Biol. 2022 Nov 14;22:529. doi: 10.1186/s12870-022-03904-2 (PMC9661794; doi:10.1186/s12870-022-03904-2)
Supplement: Supplementary file 14 — Additional file 14. [file 12870_2022_3904_MOESM14_ESM.docx]

**Table S12** The relative gene expression levels analyzed by qPCR for 15 genes in the immune systems

| Gene name | KEGG annotation | Relative expression level (fold) | | | | | |
| --- | --- | --- | --- | --- | --- | --- | --- |
|  |  | control 1 | control2 | control2 | SA1 | SA2 | SA3 |
| *Dof008571* | CNGCs | 3 | 2.1 | 2.5 | 5.9 | 3.3 | 3.8 |
| *Dof024904* | CNGCs | 8.3 | 7.3 | 7.8 | 14.1 | 14.5 | 15 |
| *Dof000577* | CDPK | 3.3 | 2.4 | 2.9 | 3.6 | 4 | 4.5 |
| *Dof010081* | CDPK | 4.1 | 3.2 | 3.6 | 6.9 | 4.4 | 4.8 |
| *Dof010899* | CDPK | 4.6 | 3.7 | 4.2 | 5.8 | 8.9 | 5.4 |
| *Dof013547* | CDPK | 2.8 | 3.3 | 3.7 | 4.4 | 3.5 | 4 |
| *Dof005640* | CaMCML | 3.9 | 4.3 | 4.8 | 5.8 | 4.9 | 5.4 |
| *Dof006104* | CaMCML | 9.6 | 10.1 | 10.5 | 14.7 | 13.8 | 14.3 |
| *Dof014321* | CaMCML | 1.4 | 1.8 | 2.3 | 3.9 | 3 | 3.4 |
| *Dof015798* | CaMCML | 3.3 | 2.4 | 2.9 | 4 | 3.1 | 3.6 |
| *Dof017381* | CaMCML | 1.9 | 1 | 1.5 | 1.6 | 0.7 | 1.1 |
| *Dof004597* | PBS1 | 4.1 | 3.2 | 3.7 | 4.3 | 4.7 | 5.2 |
| *Dof017452* | Pti1 | 10.1 | 9.2 | 9.6 | 10.8 | 14.2 | 11.7 |
| *Dof018039* | EDS1 | 2.1 | 1.2 | 1.6 | 1.9 | 2.4 | 2.8 |
| *Dof020138* | RPS2 | 0.4 | 0.8 | 1.3 | 1.2 | 3.6 | 2.1 |
